# Water Purification

**Published:** 1895-10

**Authors:** 


WATER PURIFICATION.—As the subject of water purification is now occupying a large share of professional attention in these days of dams and water works; and as much speculation is being indulged in by the sanitarians as to the best means of sterilizing the water to be used for domestic purposes, the following is reproduced as a suggestion, especially to the Austin authorities:

According to Dr. Dupont, a physician of Paris, writing in *Les Annales d' Hygiene Publique*, a safe and effective method of purifying water by chemical action has been discovered. Dr. Dupont notes that hitherto the methods of purifying drinking water have been by filtration and by the action of heat. Filtration is the oldest and least effective method. Even filtration through porous porcelain, the most effective substance known, can not always be trusted, especially after the apparatus has been long in use. Dr. Dupont does not assert that boiling fails to destroy noxious germs, but he says that it often leaves in the water organic matter that might be dangerous to health, and that boiling makes water less digestible by robbing it of its gases. He instances a case in which water from the Seine was found, after boiling, to contain more microbes than before.

M. Girar, director of the municipal laboratory in Paris, and Dr. Bordas, a pupil of Professor Bronardel, have recently presented to the Academy of Sciences, through the chemist Friedel, a communication on the purification of water by chemical action. The chemicals used are permanganate of lime and binoxide of manganese. The permanganate of lime, coming in contact with organic matter and micro organisms, destroys them and decomposes itself into oxygen, oxide of manganese and lime. Then, to carry off the surplus of permanganate and complete the purification, the water is poured over binoxide of manganese. Oxygen in the nascent state is thus freed, and it burns up any remaining germs. There remain, then, in the apparatus inferior oxides of manganese, which hasten to reoxidize themselves and furnish again a certain quantity of binoxide of magnanese. The water, as thus finally purified, contains a little lime in the form of a bicarbonate and traces of oxygenated water.

A very small quantity of permanganate of lime is used in this process. Not more than 1.3 grains troy to about a quart of water taken from the Seine at a point near Paris, resulted in the production of perfectly pure water as wholesome as spring water. Dr. Dupont says, that if the processes be made successful on a large scale, the question of purifying water is settled. Water containing 100,000 colonies of microbes per cubit centimeter can thus be purified, and ice placed in water with permanganate of lime, is also quickly sterilized.

In this connection, it would be interesting to inquire what would be the effect of passing a strong current of electricity through the water in its transit from the original source to the reservoirs or storage tanks into which it is proposed to pump it? If the source of the water supply,—(at Austin it is the lake made by the dam across the Colorado)— be allowed to become contaminated,—as has evidently been the case at Austin by the pleasure excursions and camping parties,—it goes without saying that the mere shifting of a part of the water to a reservoir, does not purify it. By the process of sedimentation it will in time become more or less clear, but it undergoes no purifying process; and it is under contemplation to filter it in some way, both going and coming; i. e., when being pumped into and out of the reservoir—and the *kind* of filter remains now to be determined.

Maj. T. J. Brackenridge, of Austin, suggests that if a wire be run through the pipes delivering the water into the reservoirs and kept highly charged, it would have the effect of destroying all organic life, and precipitating all impurities. If such is fact,—and theoretically it would seem to hold good,—it looks as if his suggestion would furnish a solution to the problem; it would render filtration unnecessary and thus prove an admirable economic as well as sanitary feature. It would only remain to devise the method of electrifying the water. It has been suggested that instead of a wire run through the pipes, a wire screen be placed at the discharging end of the pipe, which could be kept electrified, and that all water flowing through the pipe must necessarily come directly in contact with some part of the wire. It has been suggested again, that the pipes, being cast iron and closely jointed, can themselves be charged with the current.

We are not sufficiently acquainted with the subject to give any opinion; but it occurs to us that the suggestion is worthy of careful consideration and experimentation.

